# For the Antivivisectionists

**Published:** 1900-08

**Authors:** 


### FOR THE ANTIVIVISECTIONISTS.

THE Province of Quebec has been having an epidemic of smallpox and the board of health says that there were reported and treated since August of last year, when the first case was brought to their attention, no less than 515 cases. Of this number there was one death—that of a patient who had never been vaccinated; on the other hand, all the others who suffered from the disease had it in a very mild form and not one died. Here is a matter for the antivivisectionists to think of. Imagine—and there is room for imagination, too—how many deaths there would have been of the 515 cases had none of the patients ever been vaccinated. Let us imagine now, taking up the favorite argument of the misguided persons who place animal life above that of the human in point of value and care, that this one death in the province of Quebec was simply a coincidence; that the patient just happened to be free from vaccination and would have died anyway whether he had been vaccinated or not. Be that as it may, the fact remains that of all who were sick he was the only one who had never received the preventive treatment. Let that pass, however, and give the antivivisectionists the benefit of the doubt while we look over the results in Chicago which have just been made public. Since May, 1899, there have been admitted to the Chicago Isolation Hospital forty-nine cases of smallpox. In no case had the patient ever been vaccinated. In every case the police were the first to handle the patient and deliver him to the hospital authorities. Not a single policeman or police attendant contracted the disease. Every police officer in Chicago is compelled to submit to vaccination or he cannot remain on the force. Since 1894 there have been a great many cases of smallpox in Chicago coming from the lake boats and from outside the city. Since that date there have been only four cases of smallpox coming from the schools and they were light; every school child in Chicago must present a certificate from a physician that a successful vaccination has been done, else the child cannot enter the school. And yet the antivivisectionists declare that vaccination does not protect.

